# Rapid Detection of Five Estrogens Added Illegally to Dietary Supplements by Combining TLC with Raman Imaging Microscope

**DOI:** 10.3390/molecules27092650

**Published:** 2022-04-20

**Authors:** Xin Liang, Li Li, Yan Dong, Wei Dong, Hongxia Cui, Chunhui Xia, Tao Xu, Chaozhong Wang, Jie Zhang, Tingting Liu, Huimin Sui, Chao Gao

**Affiliations:** 1 School of Pharmacy, Qiqihar Medical University, Qiqihar 161006, China; liangxin@qmu.edu.cn (X.L.); dongyan00@hotmail.com (Y.D.); pingguoweiweiwei@126.com (W.D.); xutianfang@sohu.com (H.C.); chunhuixia1969@sohu.com (C.X.); harvey-333@163.com (T.X.); guanhong@qmu.edu.cn (J.Z.); ltting@outlook.com (T.L.); suihm_9@163.com (H.S.); gc1347025925@163.com (C.G.); 2 Qiqihar Institute for Food and Drug Control, Qiqihar 161006, China; wangchaozhongkcn@163.com

**Keywords:** Raman spectra, Raman Imaging Microscope, TLC, estrogen, dietary supplement

## Abstract

Estrogens added illegally to dietary supplements are hazardous to human health. Traditional detection and analysis methods have many limitations, and we have developed an assay that combines thin-layer chromatography with Raman imaging microscopy (TLC-RIM). The five estrogens (estrone, estradiol, estriol, ethinyl estradiol, and diethylstilbestrol) were initially separated by TLC, then detected by area scanning Raman imaging with a 532 nm laser under a microscope. Raman spectra were obtained for each estrogen, which were used for detecting estrogen illegally added to botanical dietary supplements. The LOD of each estrogen was 0.4, 1.0, 0.8, 0.2, and 0.2 mg/mL, respectively. The matrix in the real sample did not interfere with the detection of estrogens. The method was fast, sensitive, stable, specific, and reliable.

## 1. Introduction

The demand for botanical dietary supplements (BDS) originating from purely natural substances is prominently increasing due to the belief that these “natural” herbal products are harmless and do not cause the detrimental side effects of chemical drugs or their analogues [1]. To improve the efficacy of BDS, some pharmaceutical drugs are intentionally and illegally added, including chemicals prohibited by the administrative authority of the government [2,3]. These illegal products are hazardous to human health and could even threaten consumers’ lives. The chemicals that are most likely adulterated in BDS are hormones. Among them, steroid estrogens such as estrone (E1), estradiol (E2), estriol (E3), and ethinylestradiol (EE2) are often illegally added to anti-aging BDS; synthetic estrogens such as diethylstilbestrol (DES) are often added to BDS for breast cancer prevention [4,5,6]. These estrogens have a variety of therapeutic and pharmacological effects, which are mediated by the estrogen receptor and can cause serious toxic side effects if abused [7,8,9,10]. The illicit addition of estrogen is associated with many adverse health outcomes, such as breast cancer, heart disease, and stroke [11,12,13]. Therefore, it is very important and necessary to establish a rapid detection method for illegally added estrogens in BDS.

Many analytical techniques have been developed for the detection of the five estrogens (E1, E2, E3, EE2, and DES), i.e., E1 and E2 were detected accurately by UPLC–MS/MS [14,15], E1, E3, and EE2 in mixtures were measured quickly by GC-MS [16,17], and DES in water samples was detected sensitively by fluorimetry [18]. In addition, Raman spectroscopic discrimination of E2 and EE2 in water samples was also described [19]. All the methods are known for their high sensitivity and specificity [20]; however, the disadvantages of them include being of high cost, time consuming, and complicated in prelab sample preparation.

Raman spectroscopy coupled with thin-layer chromatography (TLC) detection methods, such as the combination of TLC and Raman spectra [21], the combination of TLC and surface-enhanced Raman spectra (SERS) [22,23,24,25], and the combination of TLC and surface-enhanced resonance Raman scattering (SERRS) [26,27], have been increasingly used for the rapid detection of chemical constituents illegally added to BDS. These methods are simple and fast, but the problem is that some components cannot be effectively separated by TLC, and the spectrum obtained by Raman detection on TLC may not be the spectrum of a single component, which may directly lead to a decrease in the accuracy of detection. This problem can be solved by Raman imaging.

The purpose of Raman imaging is to visualize the distribution of different components in a sample. Thus, each pixel in the image corresponds to a Raman spectrum, which can be compared with an established Raman database or spectra of reference substance to determine a specific analyte or spectral background measurements at this location [28,29]. There are two ways to perform Raman imaging, scanning imaging and wide-field imaging. Area scanning is one approach of wide-field Raman imaging, which enables the entire sample area to be illuminated with laser light, and its spatial information is obtained in one scan without relative movement between the laser and the sample [30]. In area scanning, when superimposed spectra of different components are obtained, the single spectra of these components can also be obtained by different colors in the imaging [31,32].

The main purpose of this study was to develop a thin-layer chromatography combined with Raman imaging microscopy method (TLC-RIM), which can rapidly separate and accurately detect small amounts of estrogens, such as E1, E2, E3, EE2, and DES in BDS. Estrogens were initially separated by TLC, the main spots on TLC were marked under 254 nm ultraviolet light, and estrogens were concentrated with anhydrous ethanol on TLC. Raman spectra of concentrated estrogen were obtained by area scanning Raman imaging with a 532 nm laser source under a microscope, which could be used to distinguish the five estrogens illegally added to BDS such as Ganoderma lucidum spore powder.

## 2. Materials and Methods

### 2.1. Materials

All reagents were bought from Merck Drugs & Co., Germany. Reference substances of E1 (98.0%), E2 (98.0%), E3 (99.0%), EE2 (99.0%), and DES (99.0%) were purchased from the National Institutes for Food and Drug Control (Beijing, China), and separated chromatographically using petroleum ether (AR, 99.5%), trichloromethane (AR, 99.0%), ethyl acetate (AR, 99.5%), and benzene (AR, 99.5%). Three real samples of Ganoderma lucidum spore powder were supplied by three different manufacturers (China) and extracted by using anhydrous ethanol (AR, 99.5%).

TLC of the sample was obtained through thin layer plate (Merck KGaA, Düren, Germany) made of high-performance silica gel and fluorescing additive F_254_, which is called GF_254_ thin layer plate for short; the particle size is 8 ± 2 μm, layer thickness is 0.2 ± 0.03 mm, and the carrier is aluminum.

### 2.2. Apparatus

Separated compounds on TLC were located under 254 nm by an Ultraviolet analyzer (YOKO-2F; Wuhan YOKO technology Ltd., Wuhan, China). Raman spectra and its imaging were obtained by use of a DXR™ xi Raman Imaging Microscope (Thermo Fisher Scientific, Waltham, MA, USA) with an excitation wavelength of 532 nm, a resolution of 5.0 cm^−1^, and a 10× long working distance microscope objective. The excitation power was 10 mW and the integration time was 0.5 s; number of scans was 20. The scan range was 3300~100 cm^−1^, with a 50 µm confocal pinhole DXR532 full range grating (400 line/mm). Detector was TE-cooled electron-multiplying CCD (EMCCD). Area scanning was chosen as the scanning mode, scanning area was more than 150 μm × 150 μm, and total scanning time was 20 min.

Ultra high-performance liquid chromatography–tandem mass spectrometry (UPLC–MS/MS) was operated on a Dionex UltiMate 3000 ultra-performance liquid chromatography–TSQ quantum mass spectrometer system (Thermo Fisher Scientific, Waltham, MA, USA).

### 2.3. Solutions Preparation

Reference substance solutions were prepared by dissolving each estrogen powder in anhydrous ethanol to prepare a solution of 1.0 mg/mL.

Mixture reference solution was prepared by dissolving all the estrogen powders together in anhydrous ethanol to prepare a solution in which each kind of estrogen was 1.0 mg/mL.

Real sample solutions were prepared by the following two steps. Extraction: Ganoderma lucidum spore powder (2 g/a single dose) 10.0 g was placed in a centrifuge tube, anhydrous ethanol 50.0 mL was added, extracted ultrasonically for 10 min, and purified centrifugally for 5 min and filtrated with 0.45 μm membrane. Concentration: solvent was evaporated in a water bath at 80 °C and reconstituted with 0.5 mL anhydrous ethanol.

Negative samples were samples that had been tested by Qiqihar Institute for Food and Drug Control and confirmed to be free of E1, E2, E2, EE2, and DES. The preparation method of negative sample solution was the same as that of real sample. Simulated positive samples were prepared by adding E1, E2, E3, EE2, and DES to negative samples, respectively. Simulated positive sample solution was prepared in the same way as real sample at the concentration of each reference substance (1.0 mg/mL).

### 2.4. The TLC Method

Thin-layer chromatography (TLC) is a simple and fast separation technique [32]. In our study, 10 μL of solutions of five control substances (E1, E2, E3, EE2, and DES) and their mixed solutions were spotted on GF_254_ thin layer plate (8 cm × 10 cm) at a distance of 1 cm from the bottom. The material was eluted to a distance of 8 cm in a development chamber saturated with petroleum ether–trichloromethane–ethyl acetate–benzene (2:6:2:2, *v*/*v*/*v*/*v*). The plate was removed and the solvent on the plate was naturally evaporated. Under UV irradiation at 254 nm, the main spots on the TLC could be observed.

### 2.5. The TLC-RIM Method

In this method, we focused on thin-layer chromatography coupled with Raman imaging microscopy (TLC-RIM) for rapid and specific detection of five estrogens added illegally to dietary supplements.

After preliminary separation of E1, E2, E3, EE2, and DES by TLC, main spots on TLC were observed and marked under 254 nm ultraviolet light. Then, estrogen in the spots was concentrated with anhydrous ethanol on TLC for Raman spectroscopic analysis. Finally, Raman images of estrogen was obtained by area scanning with 532 nm laser source under microscope, and Raman spectrum of estrogen was obtained from the Raman images. Raman spectrum of the test substance by the same method was obtained, and the Raman spectrum was consistent with the Raman spectrum of the corresponding reference substance.

## 3. Results and Discussion

### 3.1. TLC Separation

**Figure 1:**
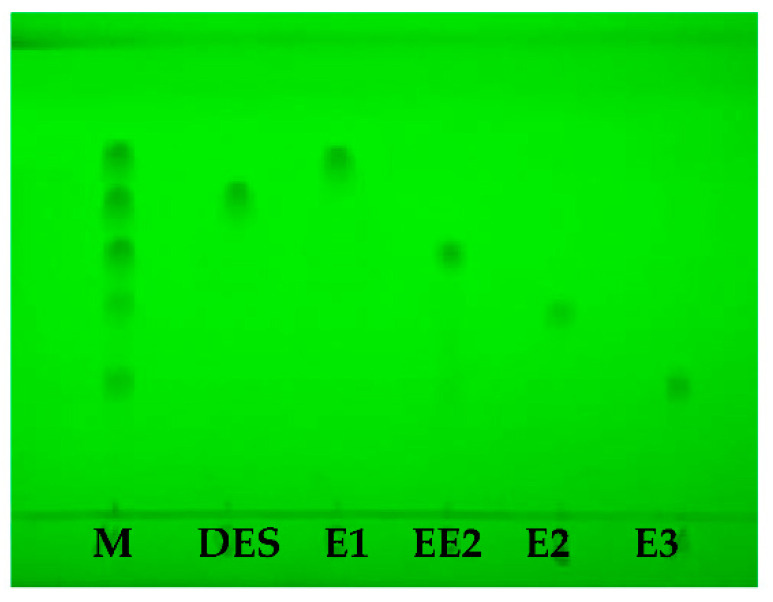
Result of five estrogens (M: mixture reference solution).

The separation of five estrogens was carried out under the condition of the TLC method as shown in Figure 1, the retardation factor (R_f_) values of E1, E2, E3, EE2, and DES were 0.73, 0.37, 0.29, 0.55, and 0.65, respectively.

### 3.2. The TLC-RIM Method

**Figure 2:**
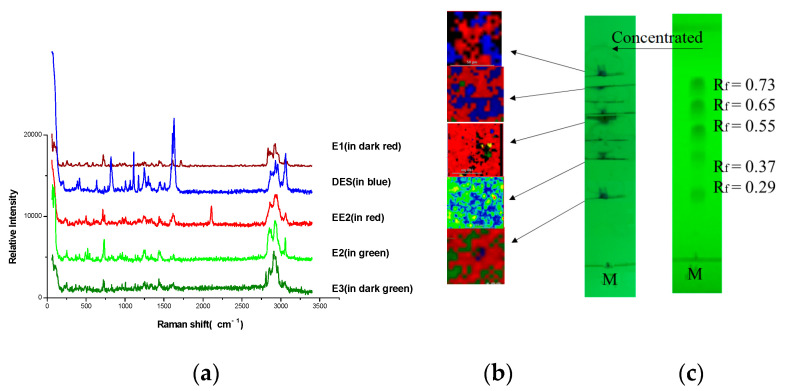
Raman spectra (**a**), imaging (**b**), and TLC (**c**) of five estrogens in mixture by TLC-RIM.

The estrogens on TLC were marked and concentrated under a 254 nm ultraviolet light, then Raman Imaging Microscope and corresponding spectra were acquired, as shown in Figure 2, respectively. The Raman spectrum of E1 was acquired as shown in the dark red region, DES in blue, EE2 in red, E2 in green, and E3 in dark green.

All the estrogens could be distinguished in the imaging and corresponding Raman spectra because Raman spectra can reflect the rich structural information of compounds. Thus, the method (TLC-RIM) used for the five estrogens was a suitable method that is very practical. The specificity could be further enhanced.

### 3.3. Correlation between Raman Spectra of Reference Powders and the Spectra by TLC-RIM

**Figure 3:**
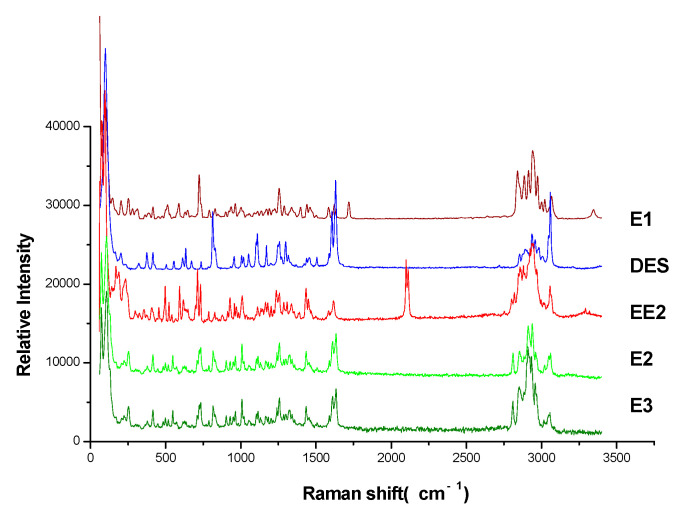
Raman spectra of estrogen powder.

The Raman spectra of estrogen powders were detected directly, the results are shown in Figure 3. The Raman spectra were observed by TLC-RIM, as shown in Figure 2a. Assignments of the Raman spectral characteristic peaks are shown in Table 1.

When the Raman spectra of reference substances detected by TLC-RIM were compared with the spectra of the corresponding powders, almost the same Raman shifts (cm^−1^) of characteristic peaks were obtained, except the relative peak intensity was slightly changed. For example, the peak intensity (735 cm^−1^, 723 cm^−1^) of E1 became weaker; the peak intensity (1619 cm^−1^, 1612 cm^−1^) of E2 became weaker too; the characteristic peaks of EE2 changed from double peaks at 2113 cm^−1^ and 2102 cm^−1^ (powder) to a single peak at 2111 cm^−1^ (TLC-RIM); the peak intensity of DES at 2960 cm^−1^, 2937 cm^−1^, 2913 cm^−1^, and 2878cm^−1^ became stronger. Therefore, the Raman spectra of estrogens acquired by the TLC-RIM method has a good correlation with the spectra of the corresponding powder.

### 3.4. Analysis of Characteristic Peaks of Estrogens

The detection results and the characteristic peaks of each estrogen are summarized in Table 1.

Common characteristic peaks: the chemical structures of five estrogens all contain methyl, methylene, and benzene rings, so there are some common characteristic peaks in their Raman spectra, such as ν_CH_ (2960~2853 cm^−1^), ν_=CH_ within phenyl rings (3060 cm^−1^), ν_C=C_ within phenyl rings (1622~1586 cm^−1^), and γ_=C-H_ within phenyl rings (821~716 cm^−1^).

The structures of E1, E2, E3, and EE2 are similar: they are all steroid hormones, except DES. There are three substituents on the benzene ring (1, 2, 4-substitution) of the steroid hormones, and two related peaks (733~716 cm^−1^) of the =CH stretching vibration in the Raman spectra. There are two substituents on the benzene ring (para-substitution) of DES, and only one related peak (821~819 cm^−1^) of the =CH stretching vibration. The steroid hormones have a higher number of -CH (methyl, methylene) bonds and lower number of =CH bonds than DES, so the peak intensity of the steroid hormones at wavenumber 2964~2853 cm^−1^ is higher than that at 3060 cm^−1^, while DES is the opposite. In addition, there is only one benzene ring in steroid hormones, so its peak heights (ν_-C=C_ within phenyl rings) are lower; there are two benzene rings in the structure of DES, so their peak heights are higher.

Unique characteristic peaks: The peak from ν_C=O_ at 1709 cm^−1^ is unique to E1, the peak from ν_C≡CH_ at 2111 cm^−1^ is unique to EE2. The peak from ν_C=C_ (symmetric vibration) at 1629 cm^−1^ is a symbol of DES. Due to the existence of the C=C structure between the two benzene rings in DES, the conjugated system is extended, resulting in the peak from ν_C=C_ in a higher Raman shift (blue shift); the peak at 1629 cm^−1^ appears when the molecules are connecting with each other through phenolic hydroxyl groups; the peak becomes a very strong peak due to the resonance Raman effect.

### 3.5. Experiment of Simulated Positive Samples

**Figure 4:**
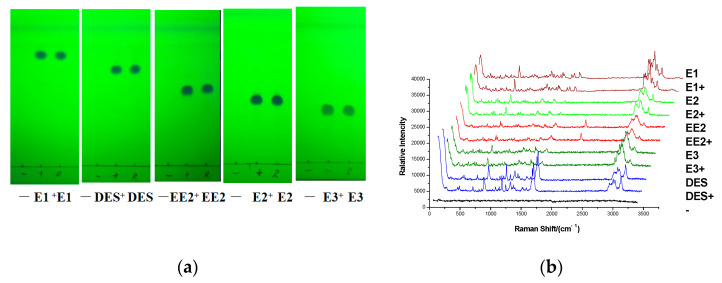
TLC (**a**) and Raman spectra (**b**) of five estrogens in simulated positive sample. (E1+, DES+, EE2+, E2+, E3+: simulated positive samples containing E1, DES, EE2, E2, and E3; −: negative sample).

Reference substance solutions, simulated positive samples, and negative sample solution of 10 μL were deposited onto the GF_254_ thin layer plate, respectively. The experiment was carried out by the TLC-RIM method, and the result was shown as follows: The major spots of the simulated positive sample were at the same position with the corresponding reference substance on TLC when no spots were observed in the negative sample on the TLC in Figure 4a. The preliminary result indicated that matrix compositions in Ganoderma lucidum spore powder did not interfere with the observing of estrogens on the TLC. At the same time, the Raman spectra of the simulated positive samples were also in accordance with the corresponding reference substances when no Raman signal was detected in the negative sample in Figure 4b. The result further confirmed that the matrix in the real sample did not interfere with the detection of estrogens that were added.

### 3.6. Detection of the Limit of Detection

**Figure 5:**
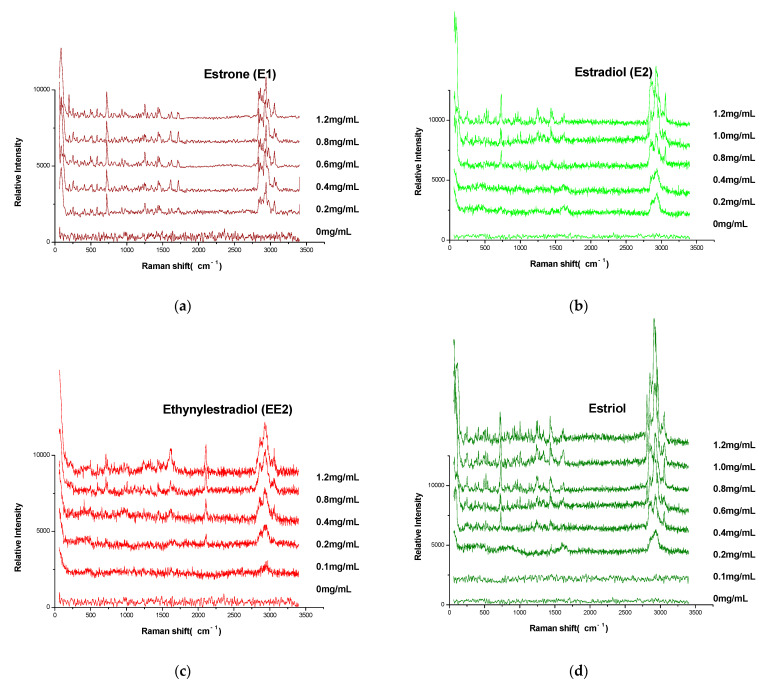

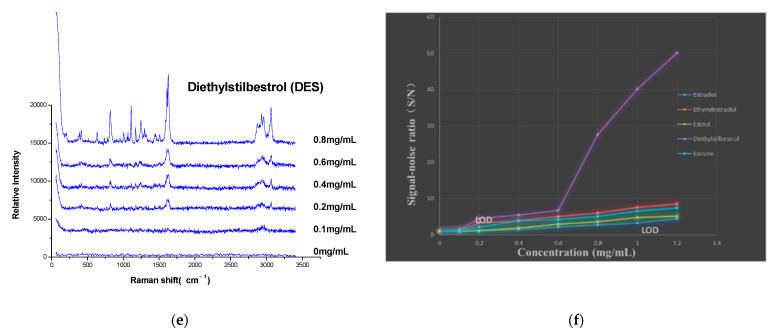
Raman spectra (**a**–**e**) and LOD (**f**) of five estrogens.

Reference substance (E1, E2, E3, EE2, and DES) solutions were diluted with anhydrous ethanol at a concentration of 0.1~1.2 mg/mL, respectively. Estrogen solutions of 10 μL of different concentrations were deposited onto GF_254_ thin layer plates, respectively, and the corresponding Raman spectra were detected; the results are shown in Figure 5. The limit of detection (LOD) was defined as the estrogen concentration for which the signal-to-noise ratio was greater than or equal to 3 (S/N ≥ 3); the signal peaks of the estrogens were: 1709 cm^−1^ (E1), 3060 cm^−1^ (E2), 2111 cm^−1^ (EE2), 3060 cm^−1^ (E3), and 1629 cm^−1^ (DES). The results are shown in Figure 5f. The LOD of E1, E2, EE2, E3, and DES were 0.4 mg/mL (S/N = 3.8), 1.0 mg/mL (S/N = 3.3), 0.2 mg/mL (S/N = 3.3), 0.8 mg/mL (S/N = 3.9), and 0.2 mg/mL (S/N = 4.5), respectively. When the solution of each concentration was detected repeatedly three times (*n* = 3), the relative standard deviation (RSD) values of each signal peak were 1.6~11.1%, 1.6~10.4%, 2.8~13.2%, 1.3~4.6%, and 0.6~10.1%, respectively.

It should be emphasized that the increase in S/N was more significant after the concentration of DES exceeded 0.6 mg/mL (Figure 5f), and the characteristic peaks of DES were significantly enhanced (Figure 5e). When the concentration was less than 0.6 mg/mL, although the S/N also increased slightly with the increase in the concentration, the increase rate was slower. The reason for this phenomenon was considered to be as follows: when the concentration of DES reaches a certain value, molecules are connected to each other through phenolic hydroxyl groups, and the conjugated chain is extended, then the resonance Raman effect results in a significant enhancement of the characteristic peaks of the Raman spectrum.

A comparison of the LOD with potentially abused estrogen doses in BDS is shown in Table 2, and the results show that the LOD is less than or equal to the potentially abused estrogen doses in BDS. Therefore, TLC-RIM is suitable for detecting estrogen that may be illegally added in BDS.

### 3.7. Detection of Real Samples

**Figure 6:**
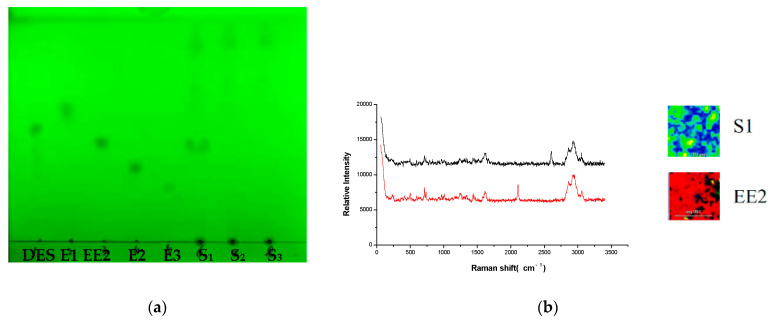
TLC (**a**) and Raman spectra (**b**) of real samples by TLC-RIM. (S_1_, S_2_, and S_3_: sample 1, sample 2, and sample 3).

A reference substance solution of 10 μL and three real sample solutions of 10 μL were deposited onto the same GF_254_ thin layer plate, respectively. There was no spot in sample 2 or sample 3 observed on the TLC in Figure 6a; none of the estrogens added illegally were found in the samples. There was only one spot in sample 1 at the same position as the reference substances (EE2) on the TLC; however, the Raman spectrum of the compound in the spot was different from EE2 in Figure 6b. The characteristic peak (2111 cm^−1^) from ν_C≡CH_ was also not found in the spectrum. This phenomenon showed that there was no EE2 added illegally found in sample 1, further proving that the specificity of the method (TLC-RIM) was stronger than that of TLC. The above experimental results of the three samples were the same as those of UPLC–MS/MS, which indicated that the method is accurate and reliable.

## 4. Conclusions

This study established a method (TLC-RIM) for the separation and detection of estrogens in BDS, which has high sensitivity, good stability, and strong specificity. In addition, the method is also simple and fast.

Raman spectra of five estrogens acquired by TLC-RIM had a good correlation with the corresponding spectra of the powder. It was shown that there are common characteristic peaks of the five estrogens, such as ν_CH_ (2960~2853 cm^−1^), ν_=CH_ within phenyl rings (3060 cm^−1^), ν_C=C_ within phenyl rings (1622~1586 cm^−1^), and γ_=C-H_ within phenyl rings (821~716 cm^−1^), and there are also unique characteristic peaks from each estrogen, such as ν_C=O_ at 1709 cm^−1^ (E1), ν_C≡CH_ at 2111 cm^−1^ (EE2), and ν_C=C_ at 1629 cm^−1^ (DES). By a simulated positive test, taking Ganoderma lucidum spore powder as an example, it was confirmed that the matrix components in a real sample would not interfere with the detection of estrogen. The experimental results of three real samples further proved that the specificity of the method (TLC-RIM) is stronger than that of TLC, and the results were verified by UPLC–MS/MS, indicating that the method is accurate and credible. In conclusion, this method can provide a new reference for the rapid detection technology research of illegally added chemical components in BDS.

## Figures and Tables

**Table 1:** Assignments of Raman spectral characteristic peaks of estrogens.

Chemical Structure	Raman Shift (cm^−1^) of Estrogen Powders (Relative Intensity)	Raman Shift (cm^−1^) of Estrogen by TLC-RIM (Relative Intensity)	Assignments
E1 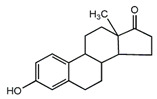	3068 (1.0) 2974, 2939 (1.8, 3.0) 2914, 2885 (2.0, 1.7) 1719 (0.8) 1590, 1615 (0.9, 1.0) 735, 723(1.4, 2.2)	3058 (1.0) 2973, 2938 (2.0, 2.2) 2915, 2883 (1.2, 1.1) 1709 (0.5) 1589, 1612 (1.0, 1.1) 732, 721(1.0, 1.7)	ν_=CH within phenyl rings_ ν^as^_CH3,_ ν^as^_CH2_ ν^s^_CH3,_ν^s^_CH2_ ν_C=O_ ν_C=C_ γ_=C-H within phenyl rings_
E2 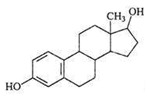	3060 (1.0) 2964, 2939 (1.0, 2.0) 2913, 2858 (2.0, 1.0) 1619, 1612 (1.7, 1.4) 732, 725 (1.2, 0.6)	3060 (1.0) 2960, 2936 (1.0, 1.6) 2911, 2853 (1.4, 1.3) 1622, 1609 (0.5, 0.4) 732, 722 (1.0, 0.4)	ν_=CH within phenyl rings_ ν^as^_CH3,_ ν^as^_CH2_ ν^s^_CH3,_ν^s^_CH2_ ν_C=C_ γ_=C-H within phenyl rings_
E3 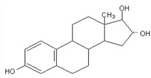	3060 (1.0) 2964, 2939 (1.0, 2.2) 2913, 2858 (1.3, 1.3) 1614 (0.7) 723, 709 (1.3, 0.7)	3060 (1.0) 2960, 2936 (1.0, 1.6) 2911, 2853 (1.2, 1.2) 1613 (0.6) 732, 722 (1.2, 0.5)	ν_=CH within phenyl rings_ ν^as^_CH3,_ ν^as^_CH2_ ν^s^_CH3,_ν^s^_CH2_ ν_C=C_ γ_=C-H within phenyl rings_
EE2 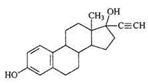	3056 (1.0) 2965, 2941 (1.4, 2.2) 2910, 2860 (1.6, 1.5) 2113, 2102 (1.4, 1.5) 1617, 1602 (0.7, 0.5) 735, 713 (1.0, 1.4)	3063 (1.0) 2957, 2935 (1.5, 2.3) 2873, 2861 (1.8, 1.8) 2111 (1.7) 1615, 1601 (0.9, 0.6) 733, 716 (0.7, 1.2)	ν_=CH within phenyl rings_ ν^as^_CH3,_ ν^as^_CH2_ ν^s^_CH3,_ν^s^_CH2_ ν_C__≡C_ ν_C=C_ γ_=C-H within phenyl rings_
DES 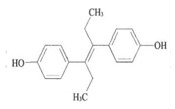	3058 (1.0) 2960, 2937 (0.3, 0.4) 2913, 2878 (0.3, 0.2) 1633 (1.2) 1608, 1600 (0.7, 0.1) 817 (0.8)	3061 (1.0) 2960, 2930 (0.8, 0.7) 2902, 2870 (0.6, 0.6) 1629 (1.4) 1608, 1593 (1.2, 0.8) 821 (1.0)	ν_=CH within phenyl rings_ ν^as^_CH3,_ ν^as^_CH2_ ν^s^_CH3,_ν^s^_CH2_ ν_C=C symmetric vibration_ ν_C=C within phenyl rings_ γ_=C-H within phenyl rings_

ν, stretching vibration; γ, out-of-plane bending vibration. Calculated the relative intensities of other peaks with the peak of 3061 cm^−1^ as reference peak. The unique peaks are marked in red, and the peaks marked in blue are those whose relative intensity or number of peaks had changed when measured by TLC-RIM method.

**Table 2:** The comparison of LOD with potentially abused estrogen doses in BDS.

Drugs	The minimum Therapeutic Dosage in Oral Medications (mg/a Day)	Potentially Abused Dosages of Drugs in BDS (mg/a Single Dose)	The Possible Concentration of the Drug in Sample Solution (mg/mL)	LOD (mg/mL)
E1	1	1	10	0.4
E2	1	1	10	1.0
E3	1	1	10	0.8
EE2	0.02	0.02	0.2	0.2
DES	0.25	0.25	2.5	0.2

## Data Availability

Not applicable.

## Sample Availability

Samples of the compounds are available from the authors.
